# Association of statin use and clinical outcomes in heart failure patients: a systematic review and meta-analysis

**DOI:** 10.1186/s12944-019-1135-z

**Published:** 2019-10-31

**Authors:** Agata Bielecka-Dabrowa, Ibadete Bytyçi, Stephan Von Haehling, Stefan Anker, Jacek Jozwiak, Jacek Rysz, Adrian V. Hernandez, Gani Bajraktari, Dimitri P. Mikhalidis, Maciej Banach

**Affiliations:** 1 Department of Hypertension, Medical University of Lodz, Rzgowska, 281/289; 93-338 Łódź, Poland; 2 Department of Cardiology and Congenital Diseases of Adults, Polish Mother’s Memorial Hospital Research Institute (PMMHRI), Lodz, Poland; 3 Clinic of Cardiology, University Clinical Centre of Kosovo, Prishtina, Republic of Kosovo; 4 Department of Public Health and Clinical Medicine, Umeå University, Umeå, Sweden; 5 Department of Cardiology and Pneumology, University Medical Center Gottingen (UMG), Gottingen, Germany; 6 Charité-Universitätsmedizin Berlin, Berlin, Germany; 7 Department of Family Medicine and Public Health, Institute of Medicine, University of Opole, Opole, Poland; 8 Department of Nephrology, Hypertension and Family Medicine, Medical University of Lodz, Lodz, Poland; 9 Health Outcomes, Policy, and Evidence Synthesis (HOPES) Group, University of Connecticut School of Pharmacy, Storrs, CT USA; 10 School of Medicine, Universidad Peruana de Ciencias Aplicadas (UPC), Lima, Peru; 11 Department of Clinical Biochemistry, Royal Free Campus, University College London Medical School, University College London (UCL), London, UK

**Keywords:** Statins, Heart failure, Mortality, Hospitalization, Meta-analysis

## Abstract

**Background:**

The role of statins in patients with heart failure (HF) of different levels of left ventricular ejection fraction (LVEF) remains unclear especially in the light of the absence of prospective data from randomized controlled trials (RCTs) in non-ischemic HF, and taking into account potential statins’ prosarcopenic effects. We assessed the association of statin use with clinical outcomes in patients with HF.

**Methods:**

We searched PubMed, EMBASE, Scopus, Google Scholar and Cochrane Central until August 2018 for RCTs and prospective cohorts comparing clinical outcomes with statin vs non-statin use in patients with HF at different LVEF levels. We followed the guidelines of the 2009 PRISMA statement for reporting and applied independent extraction by multiple observers. Meta-analyses of hazard ratios (HRs) of effects of statins on clinical outcomes used generic inverse variance method and random model effects. Clinical outcomes were all-cause mortality, cardiovascular (CV) mortality and CV hospitalization.

**Results:**

Finally we included 17 studies (n = 88,100; 2 RCTs and 15 cohorts) comparing statin vs non-statin users (mean follow-up 36 months). Compared with non-statin use, statin use was associated with lower risk of all-cause mortality (HR 0.77, 95% confidence interval [CI], 0.72–0.83, P < 0.0001, I^2^ = 63%), CV mortality (HR 0.82, 95% CI: 0.76–0.88, *P* < 0.0001, I^2^ = 63%), and CV hospitalization (HR 0.78, 95% CI: 0.69–0.89, P = 0.0003, I^2^ = 36%). All-cause mortality was reduced on statin therapy in HF with both EF < 40% and ≥ 40% (HR: 0.77, 95% Cl: 0.68–0.86, P < 0.00001, and HR 0.75, 95% CI: 0.69–0.82, *P* < 0.00001, respectively). Similarly, CV mortality (HR 0.86, 95% CI: 0.79–0.93, *P* = 0.0003, and HR 0.83, 95% CI: 0.77–0.90, *P* < 0.00001, respectively), and CV hospitalizations (HR 0.80 95% CI: 0.64–0.99, *P* = 0.04 and HR 0.76 95% CI: 0.61–0.93, *P* = 0.009, respectively) were reduced in these EF subgroups. Significant effects on all clinical outcomes were also found in cohort studies’ analyses; the effect was also larger and significant for lipophilic than hydrophilic statins.

**Conclusions:**

In conclusion, statins may have a beneficial effect on CV outcomes irrespective of HF etiology and LVEF level. Lipophilic statins seem to be much more favorable for patients with heart failure.

## Background

The management of heart failure (HF) remains a significant challenge. The most recent American College of Cardiology/American Heart Association (ACC/AHA) guidelines on the treatment of blood cholesterol have no recommendation regarding statin therapy in patients with New York Heart Association class II-IV HF [1]. Moreover, the recent European Society of Cardiology (ESC) guidelines for the diagnosis and treatment of HF do not support the initiation of statin therapy in most patients with chronic HF and reduced left ventricular (LV) ejection fraction (HFrEF). However, in HF patients who already are under the treatment with statin therapy because of underlying coronary artery disease (CAD) and/or hyperlipidemia, continuation of this therapy should be considered [2].

The issue of whether or not to use statins in patients with HF remains controversial. More than half of patients with HF have LV mid-range EF (HFmrEF) and preserved LVEF (HFpEF) and mortality and morbidity of patients with these types of HF are also high [3, 4]. The pathophysiology of HFpEF is poorly understood, and the presence of a systemic pro-inflammatory state was also proposed [3, 4].

Statins (3-hydroxy-3-methyl-glutaryl-CoA reductase inhibitors), apart from their lipid-lowering properties and mevalonate inhibition, exert their actions through multiple additional mechanisms [5]. These pleiotropic effects of statins may potentially influence the course of HF. Therefore, the aim of this meta-analysis was to assess the effect of statins on clinical outcomes in patients with HF.

## Methods

We followed the guidelines of the 2009 PRISMA statement [6] for reporting. Due to the study design (meta-analysis), neither Institutional Review Board (IRB) approval nor patient informed consent was needed.

### Search strategy

We searched PubMed, EMBASE, Scopus, Google Scholar, the Cochrane Central Registry of Controlled Trials and ClinicalTrial.gov until August 2018, using the following keywords: ‘heart failure’ OR ‘HF’ OR ‘left ventricular dysfunction’ OR ‘heart failure with preserved ejection fraction’ OR ‘HFpEF’ ‘heart failure with reduced ejection fraction’ OR ‘HFrEF’ OR ‘heart failure with mid-range ejection fraction’ OR ‘HFmrEF’ AND ‘statin’ OR ‘statins’ OR ‘lipid-lowering therapy’ OR ‘dyslipidemia therapy’ OR ‘simvastatin’ OR ‘atorvastatin’ OR ‘rosuvastatin’ OR ‘pitavastatin’ OR ‘pravastatin’ OR ‘lovastatin’ AND ‘all-cause mortality’, ‘cardiovascular mortality’, ‘hospitalizations’ AND ‘lipid’ OR ‘lipids’ OR ‘cholesterol’ OR ‘lipoprotein’ OR ‘lipoproteins’. The details on the search strategy can be found in the Additional file 1. Additional searches for potential trials included the references of review articles, and abstracts at ESC, AHA, ACC, European Society of Atherosclerosis (EAS) and National Lipid Association (NLA) meetings. The literature search was limited to articles published in English and to studies in humans.

### Study selection

We included randomized controlled trials (RCTs) and prospective cohort studies with HF patients with LVEF < 40% and ≥ 40%, i.e. involving all types of patients as per the 2016 ESC HF guidelines classification: preserved, mid-range and reduced ejection fraction (HFpEF, HFmrEF and HFrEF) [7]. Because most of studies were performed before 2016, we divided them into HFrEF studies (patients with LV EF < 40%) and both HFpEF and HFmrEF studies (patients with EF ≥40%). Other inclusion criteria were: follow-up ≥12 months, CV events as the primary or secondary outcomes, a control arm, ≥50 participants, and patients of 18 years or older.

Exclusion criteria were: (1) retrospective studies (2), follow-up < 12 months, and (3) ongoing trials. Two reviewers (AB-D and IB) independently evaluated each article separately. No filters were applied. The remaining articles were obtained in full-text and assessed again by the same two researchers. Disagreements were resolved by discussion with a third party (MB).

### Outcome variables

Primary clinical outcomes were: all-cause mortality, cardiovascular (CV) mortality and CV hospitalization. We used study definitions for all outcomes. We evaluated the longest available follow-up according to per protocol definitions.

### Data extraction

We independently extracted: 1) first author’s name, 2) year of publication, 3) name of study, 4) country where the study was performed, 5) number of centers, 6) study design, 7) number of participants per arm 8) HF and statin, 9) mean follow-up, 10) age and sex of study participants, 10) baseline level of triglycerides (TGs) and total cholesterol (TC), 11) diabetes mellitus (DM) and arterial hypertension (HTN), and, 12) data regarding CV events. Discrepancies in extractions were resolved by discussion with a third author (MB).

### Risk of bias assessment

Assessment of risk of bias RCTs was evaluated by the same investigators for each study and was performed independently using the Cochrane risk of bias tool [8]. Evaluated items were: random sequence generation (selection bias), allocation sequence concealment (selection bias), blinding of participants and personnel (performance bias), blinding of outcome assessment (detection bias), incomplete outcome data (attrition bias), selective outcome reporting (reporting bias) and other potential sources of bias. The risk of bias in each study was judged to be “low”, “high” or “unclear”.

For the assessment of risk of bias in cohort studies we used the Newcastle-Ottawa Scale (NOS). Three domains were evaluated with the following items: a. Selection: 1) representativeness of the exposed cohort, 2) selection of the non-exposed cohort, 3) ascertainment of exposure and 4) demonstration that outcome of interest was not present at start of study; b. Comparability of exposed and non-exposed; and c. Exposure: 1) assessment of outcome, 2) was follow-up long enough for outcomes to occur?, and 3) adequacy of follow-up of cohorts. The risk of bias in each study was judged to be “good”, “fair” or “poor” [9].

### Statistical analysis

A two-tailed p < 0.05 was considered significant [10]. Study baseline characteristics were reported as median and range. Mean and standard deviation (SD) values were estimated using the method described by Hozoetet et al. [11]. Meta-analyses were performed with random effects models as we expected heterogeneity of effects among studies. The generic inverse variance method was used to combine log hazard ratios (log HR) and standard errors of the log HR (SElogHR). The log HRs were adjusted for a common set of confounders across studies, such as age and gender. Heterogeneity between studies was assessed using the Cochrane Q test and I^2^statistic. As a guide, I^2^ < 25% indicated low, 25–50% moderate and > 50% high heterogeneity [12]. Publication bias was assessed using visual inspections of funnel plots and Egger’s test. Subgroup analyses by EF level (< 40% vs ≥ 40%) and type of statin (lipophilic vs hydrophilic) were performed. Sensitivity analyses in cohort studies only were also done. Meta-analyses were conducted using RevMan 5.1 (The Cochrane Collaboration, Copenhagen, Denmark).

## Results

### Search results and trial flow

**Fig. 1:**
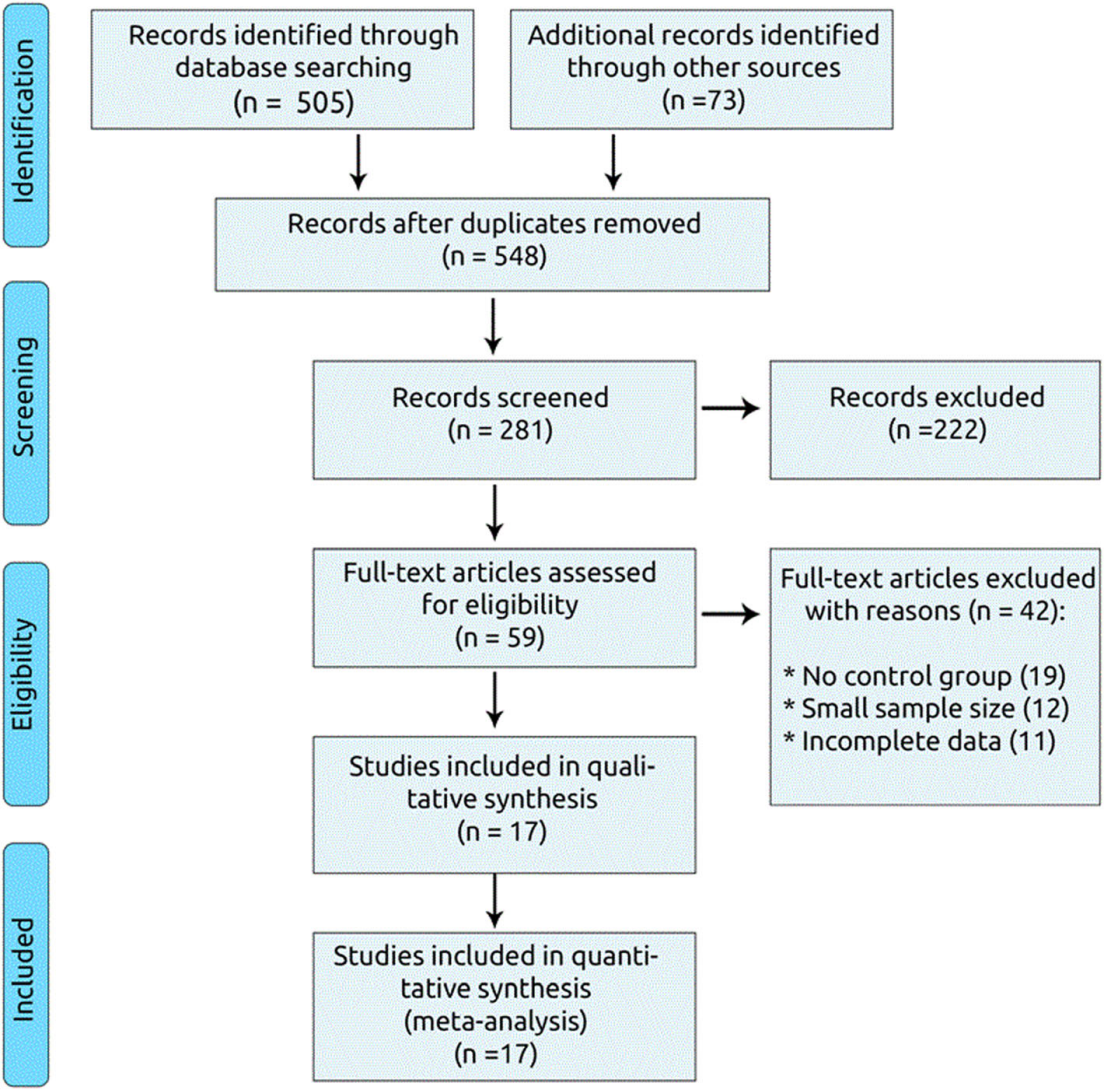
Flow chart of studies

Of 578 articles initially identified, 281 studies were screened as potentially relevant. After excluding 222 studies, 59 full text articles were assessed. Among the remaining 59 trials checked for eligibility, 42 studies were excluded. After careful assessment, 17 articles met the inclusion criteria [13–29]: two RCTs (n = 9585) and 15 cohort studies (*n* = 78,515) (Fig. 1).

### Characteristics of included studies

Seventeen studies with a total of 88,100 patients, 42,400 treated with statins and 45,700 without statins, with a mean follow-up 36 months were finally included in the meta-analysis (Table 1). The mean age of patients was 67 ± 7.2 years, 68% male, 33% had diabetes, 71% had arterial hypertension and 54% were smokers (Table 2). Studies of Sola (2005) [14], Go (2006) [17], Roik (2008) [21], and Hong (2005) [16] evaluated lipophilic statins and studies of Kjekshus [18] (2007), Tavazzi (2008) [22] hydrophilic statins.

**Table 1:** Main characteristics of studies included in the study

Study, year	Study design	Type of HF	Inclusion Criteria	Exclusion Criteria	Study comparison	Type of statins	Primary endpoints	Follow-up
Horwich et al. 2004	Prospective cohort	HFrEF	HF patients	EF > 40% Baseline incomplete data	Statins: Control	Not specified	All-cause mortality; mortality mortality	12 mo
Sola et al. 2005	Prospective cohort	HFrEF	HF patients EF ≤35% NYHA II-III	Prescribed statins > 1 year; intolerance to statins	Statins: Control	Atorvastatin Fluvastatin Pitavastatin Simvastatin	All-cause mortality; Hospitalization	24 5 mo
Fukuta et al. 2005	Prospective cohort	HFpEF	HF patients	EF < 50% Significant valvular disease; prosthetic valve	Statins: Control	Atorvastatin Simvastatin Pravastatin Fluvastatin	All-cause mortality; Hospitalizations	21 ± 12 mo
Hong et al. 2005	Prospective cohort	HFrEF	HF patients < 40%	HF patients with EF > 40%	Statins: Control	Simvastatin		12 mo
Go et al. 2006	Prospective cohort	HFrEF	HF patients		Statins: Control	Not specified	All-cause mortality; Hospitalizations	28 mo
Kjekshus et al. 2007 CORONA	RCTs	HFrEF	HF patients, EF < 40%, NYHA II-IV	previous statin- induced myopathy or hypersensitivity decompensated HF	Statins: Control	Rosuvastatin	CV death Non-fatal MI Stroke	38.2 mo
Huan et al. 2007	Prospective cohort	HFrEF	HF patients with LVSD	HF patients with LVDD	Statins: Control	Not specified	All-cause mortality;	36 mo
Coleman et al. 2008	Prospective cohort	HFrEF	HF patients EF < 40%,und--ergoing ICD		Statins: Control	Not specified	All-cause Mortality VT/VF incidence	31 mo
Roik et al. 2008	Prospective cohort	HFpEF	HF patients with preserved EF	LVEF ≤45%, ACS cardiogenic shock severe AS, etc.	Statins: Control	Simvastatin Atorvastatin	All-cause mortality; Hospitalization	12 mo
Tevazzi et al. 2008 the GISSI-HF trial	RCTs	HFrEF	HF patients NYHA II-IV	Non-cardiac comorbidity (cancer)	Statins: Control	Rosuvastatin	All-cause mortality; Hospitalization	3.9 y
Gomez-Soto et al. 2010	Prospective cohort	HFpEF	HF patients with preserved EF	HF patients with reduced EF	Statins: Control	Not specified	All-cause mortality; CV mortality Hospitalization	34.6 mo
Kaneko et al. 2013	Prospective cohort	HFpEF	HF patients with EF ≥50%	Valvular heart disease EF < 50%	Statins: Control	Not specified	CV mortality Hospitalization	3 y
Yap et al. 2015	Prospective cohort	HFpEF	HF patients with EF ≥50%	Incomplete follow-up Non-documented EF	Statins: Control	Not specified	All-cause mortality;	2 y
Nochioka et al. 2015	Prospective cohort	HFpEF	HF patients with stages B-D	NR	Statins: Control	Not specified	All-cause mortality; Hospitalization	3 y
Alehagen U et al. 2015	Prospective cohort	HFpEF	HF patients with EF ≥50%	HF patients with EF < 50%	Statins: Control	Not specified	All-cause mortality;	12 mo
Alehagen et al. 2015	Prospective cohort	HFrEF	HF patients	HF patients with EF ≥50%	Statins: Control	Not specified	All-cause mortality;	24 mo
Tsujimoto et al. 2018	Prospective cohort	HFpEF	HF patients with preserved EF	HOCMP systemic illness with l Life expectancy < 3 y;			All-cause mortality; CV and Non-CV mortality;	3.3 y

Abbreviations: HF: heart failure; HFrEF: heart failure with reduced ejection fraction; HFpEF: heart failure with preserved ejection fraction; CV: cardiovascular; ACS: acute coronary syndrome; AS: aortic stenosis; NR: non-reported; mo: months; y: years

**Table 2:** Main characteristics of patients enrolled among trials included in the meta-analysis

Study, year	Arms	No	EF %	Age Year	BMI	Male %	DM %	HTN %	Smoking %	TC mmol/L	Triglyceride mmol/L
Horwich et al. 2004	S C	200 250	≤40% ≤40%	57 ± 11 48 ± 13	28.2 ± 6.2 26.9 ± 6.2	82 70	33 16	64 43	80 66	4.32 ± 1.25 4.2 ± 1.5	1.87 ± 1.3 1.98 ± 2.17
Sola et al. 2005	S C	225 191	≤35% ≤35%	55.4 ± 6.4 53.8.4 ± 5.7	24.3 ± 3.8 23.5 ± 4.3	62 63	24 27	41 36	34 30	NR NR	2.8 ± 0.5 2.9 ± 0.4
Fukuta et al. 2005	S C	69 68	≥50% ≥50%	65 ± 2 65 ± 16	NR NR	51 45	34 12	87 72	NR NR	6.07 ± 2.22 4.67 ± 1	2.31 ± 2.21 1.64 ± 0.90
Hong et al. 2005	S C	106 96	≤40% ≤40%	61.8 ± 10.3 60.9 ± 10.4	NR NR	72 75	32 28	41 44	57 52	NR NR	NR NR
Go et al. 2006	S C	12,648 11,950	≤40% ≤40%	69.6 ± 10.3 72.9 ± 11.4	NR NR	62 60	55.7 41.3	89 83	NR NR	5.37 ± 1.14 5.68 ± 1.22	NR NR
Kjekshus et al. 2007	S C	2514 2497	≤40% ≤40%	73 ± 7.1 73 ± 7.0	27 ± 4.5 27 ± 4.6	76 76	30 29	63 63	9 8	5.36 ± 1.11 5.35 ± 1.06	2.01 ± 1.33 1.99 ± 1.23
Huan et al. 2007	S C	377 102	≤40% ≤40%	74 ± 4 74 ± 3	26.5 28.1	66 77	NR NR	NR NR	75 72	5.1 ± 0.25 5.5 ± 0.3	NR NR
Coleman et al. 2008	S C	642 562	≤30% ≤30%	67.5 ± 13 64.5 ± 10.8	NR NR	80.7 76.2	31.5 30.2	43.8 34.9	NR NR	NR NR	NR NR
Roik et al. 2008	S C	103 43	≥45% ≥45%	69 ± 11 66 ± 16	28.6 ± 4.8 27.2 ± 4.9	50.5 58	25 12	76 58	43 34	4.57 ± 1.37 4.57 ± 1.03	1.64 ± 1.08 1.62 ± 1.05
Tavazzi et al. 2008 the GISSI-HF trial	S C	2285 2289	33.4 33.4	68 ± 1 68 ± 1	27·1 ± 4.6 27.71 ± 4.4	78.6 76.8	25 27.4	53.5 55.1	14.1 14	NR NR	NR NR
Gomez-Soto et al. 2010	S C	1343 1230	≥47% ≥47%	71.5 ± 6.9 69.8 ± 7.8	NR NR	51.6 43.9	36.8 47.7	45.6 48.5	33 31	NR NR	NR NR
Kaneko et al. 2013	S C	459 665	≥50% ≥50%	65.6 ± 11.7*	24.3 ± 3.6*	76.2*	32.4*	64.5*	24.2*	NR NR	NR NR
Yap et al. 2015	S C	457 293	≥50% ≥50%	73.1 ± 10.6*	26.5*	35.3*	47.1*	80.3*	NR NR	NR NR	NR NR
Nochioka et al. 2015	S C	1163 1961	≥50% ≥50%	69.0 ± 11.0 69.7 ± 12.9	67.5 64	45 40.8	33.8 20.9	85 76.7	45 40.8	NR NR	1.51 ± 0.82 1.4 ± 0.81
Alehagen U et al. 2015	S C	3427 5713	≥50% ≥50%	78 ± 12 75 ± 9	29 ± 6 27 ± 6	54 42	31 28	62 64	NR NR	NR NR	NR NR
Alehagen et al. 2015	S C	10,345 11,519	< 40% < 40%	72 10 72 ± 14	27 ± 5 26 ± 5	75 68	33 18	48 39	41 49	NR NR	NR NR
Tsujimoto et al. 2018	S C	1765 1613	≥50% ≥50%	69 ± 9.6 68.1 ± 9.6	NR NR	55 42	42.9 20.8	93.1 89.8	10.5 10.6	NR NR	NR NR

Abbreviations: S: statins; C: control; HTN: hypertension; DM: diabetes mellitus; TC: total cholesterol; EF: ejection fraction; NR: not-reported; *: only whole group represented

### Clinical outcomes

**Fig. 2:**
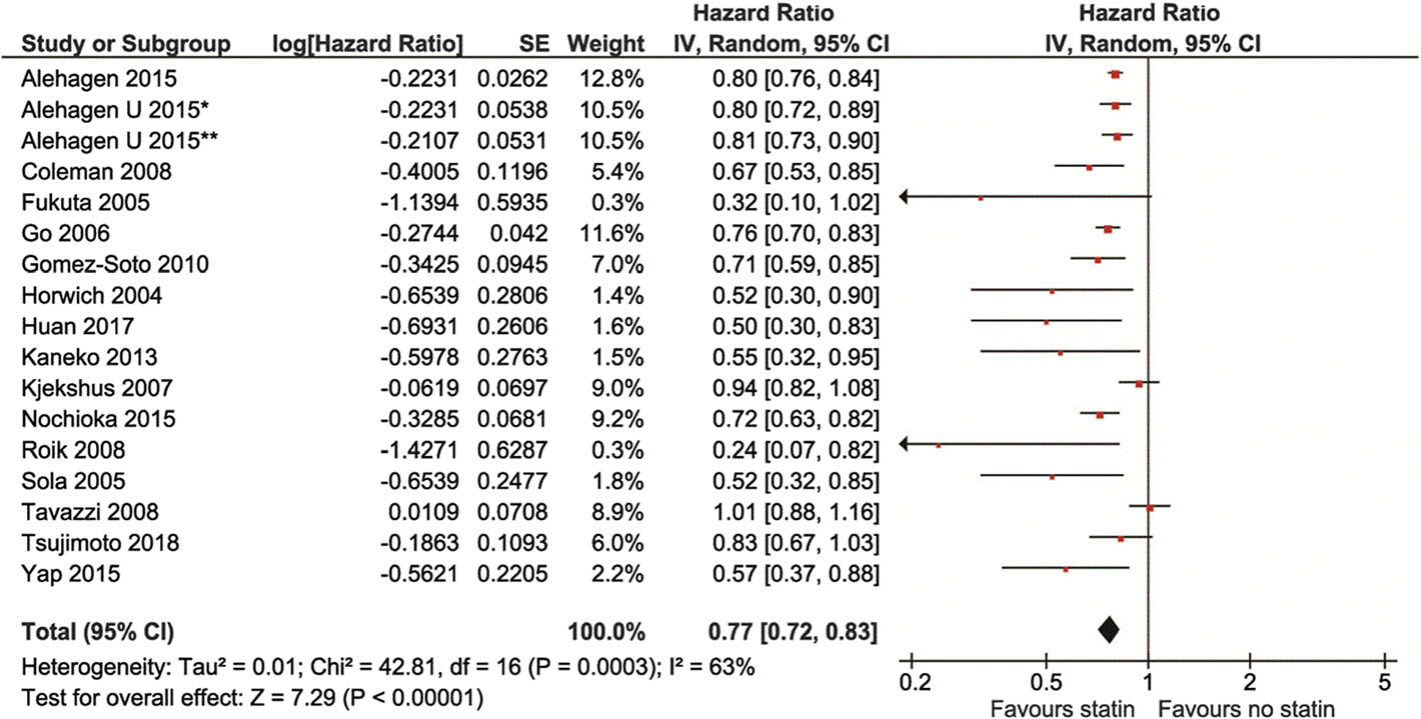
Association of statin versus non-statin use with all-cause mortality in heart failure

**Fig. 3:**
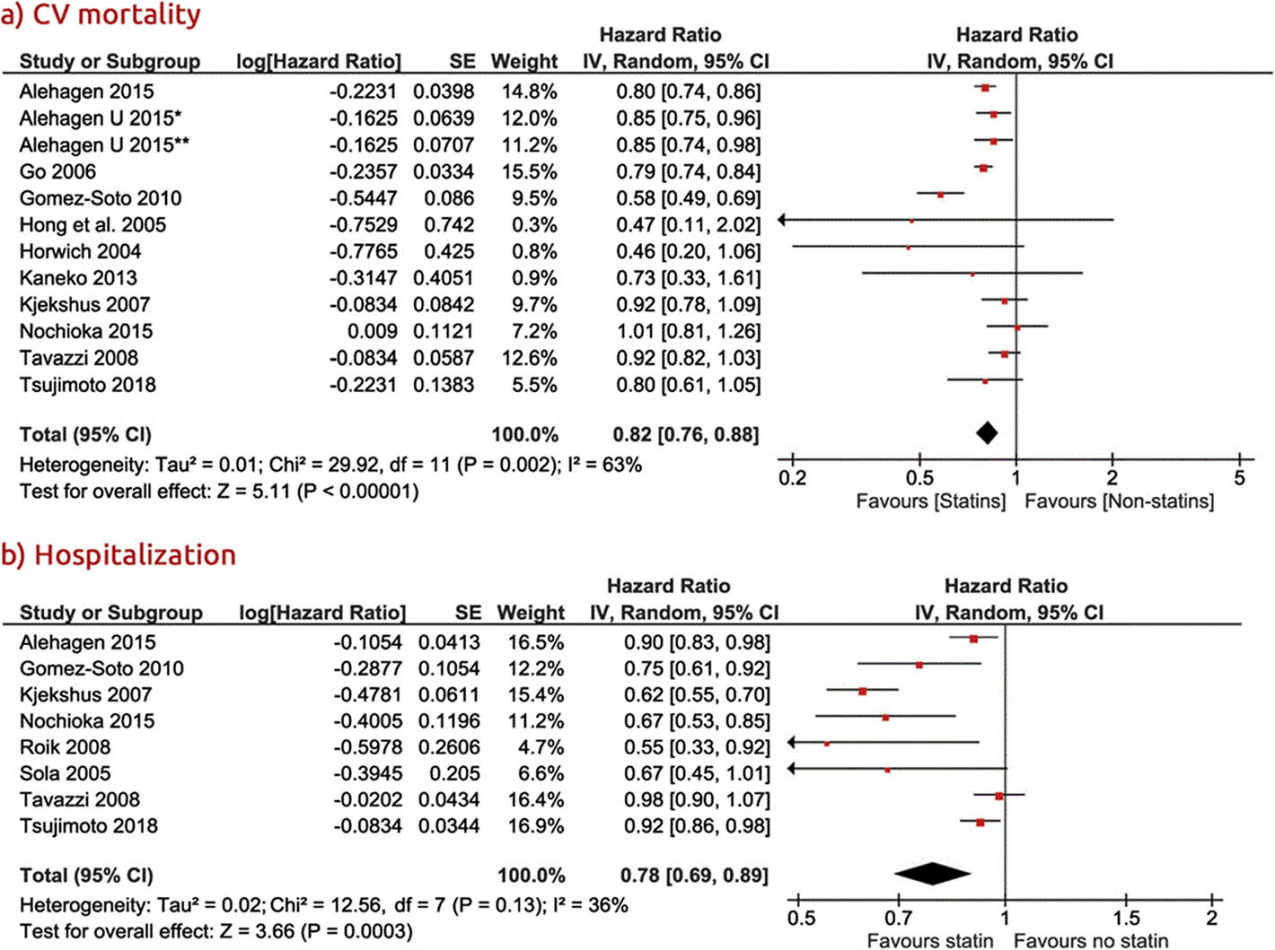
Association of statin versus non-statin use with **a**) CV mortality, and **b**) CV hospitalizations

Follow-up ranged from 12 to 40 months, with a mean of 36 months. Compared with non-statin users, statin users showed a lower risk of all-cause mortality (HR 0.77, 95% confidence interval [CI], 0.72–0.83, *P* < 0.0001, I^2^ = 63%, Fig. 2), CV mortality (HR 0.82, 95% Cl: 0.76–0.88, *P* < 0.000, I^2^ = 63%) and CV hospitalization (HR 0.78, 95% Cl: 0.69–0.89, *P* = 0.0003, I^2^ = 36%, Fig. 3a and b).

### Subgroup analyses

**Fig. 4:**
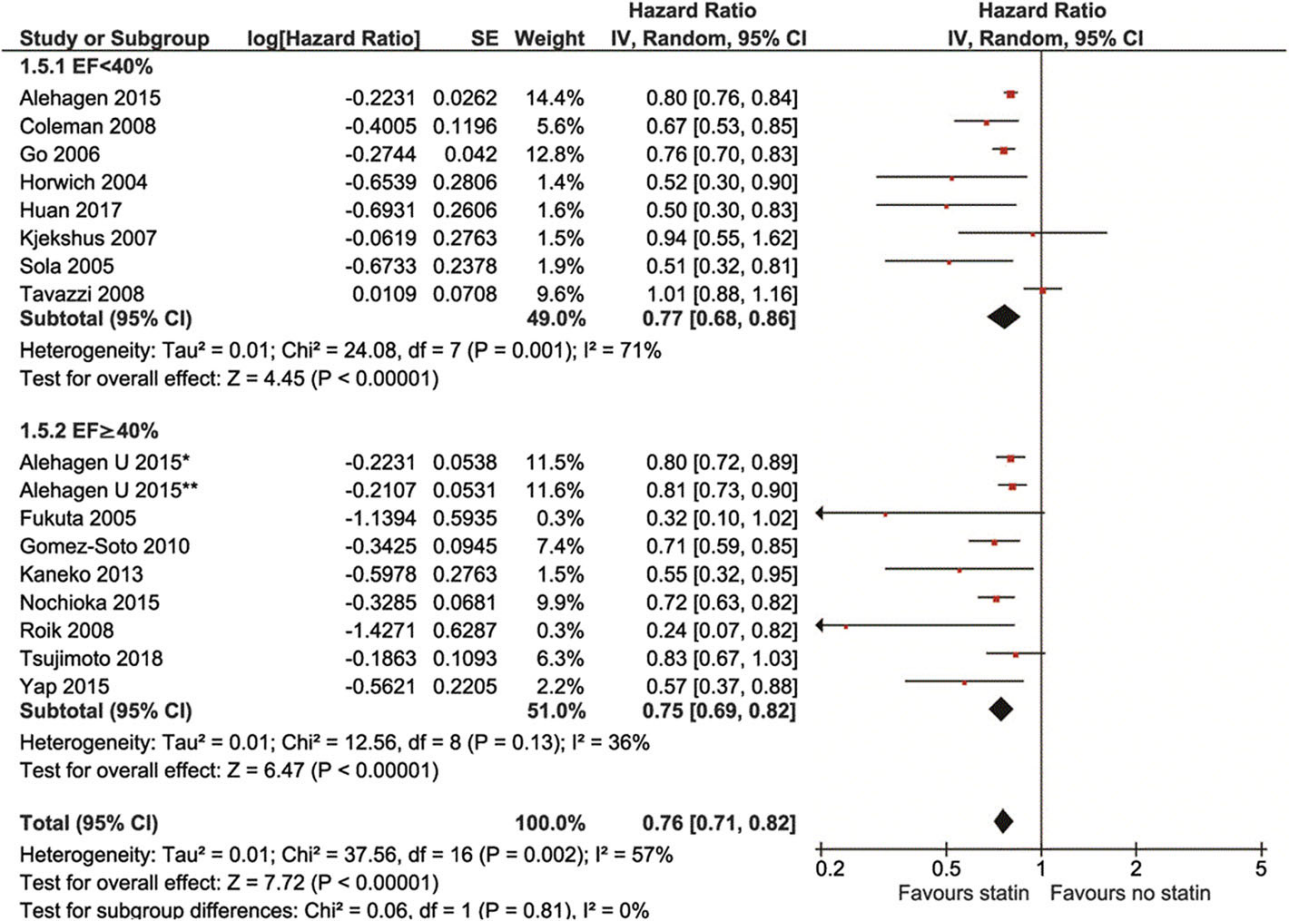
Association of statin versus non-statin use with all-cause mortality by type of heart failure

**Fig. 5:**
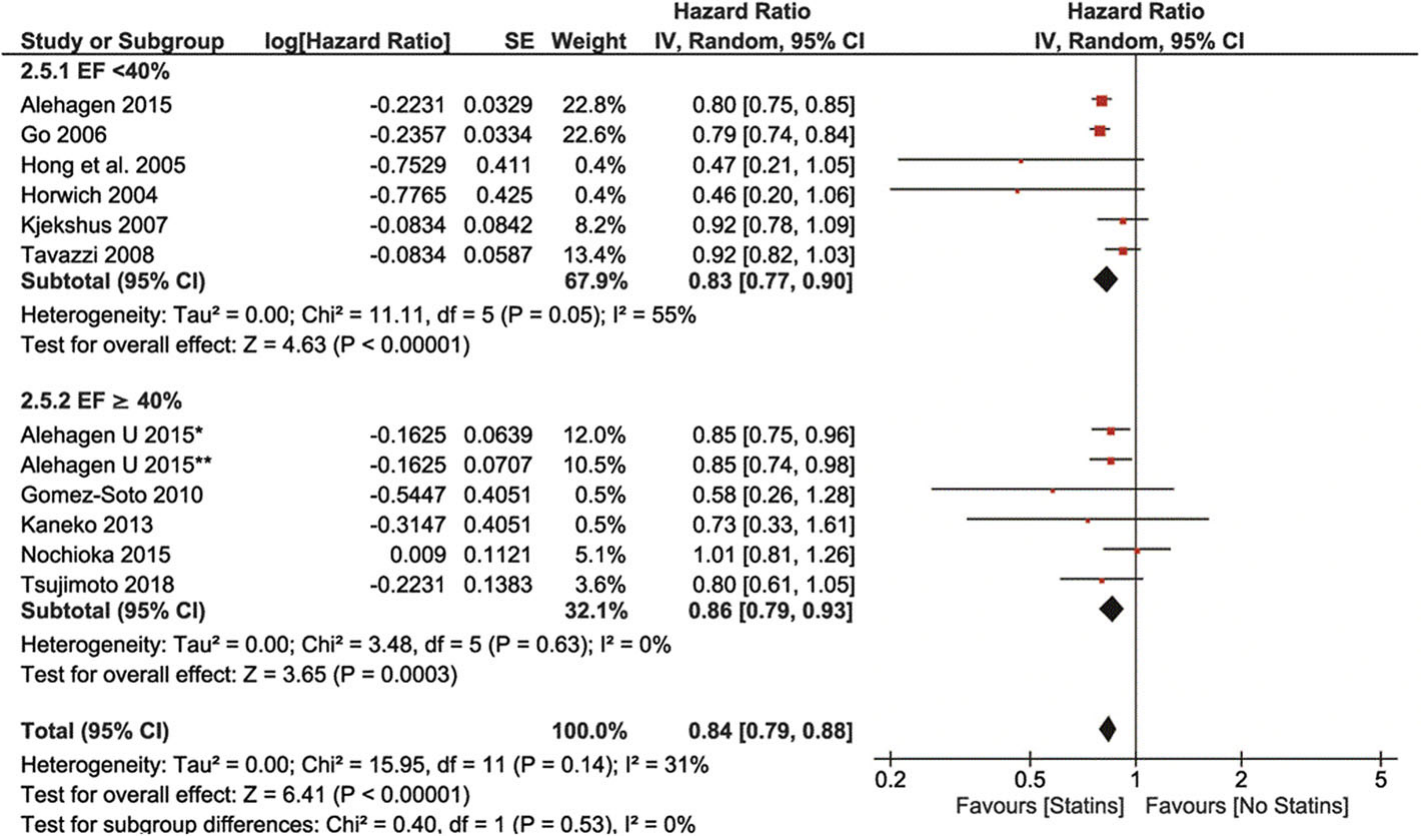
Association of statin versus non-statin use with CV mortality by type of heart failure

**Fig. 6:**
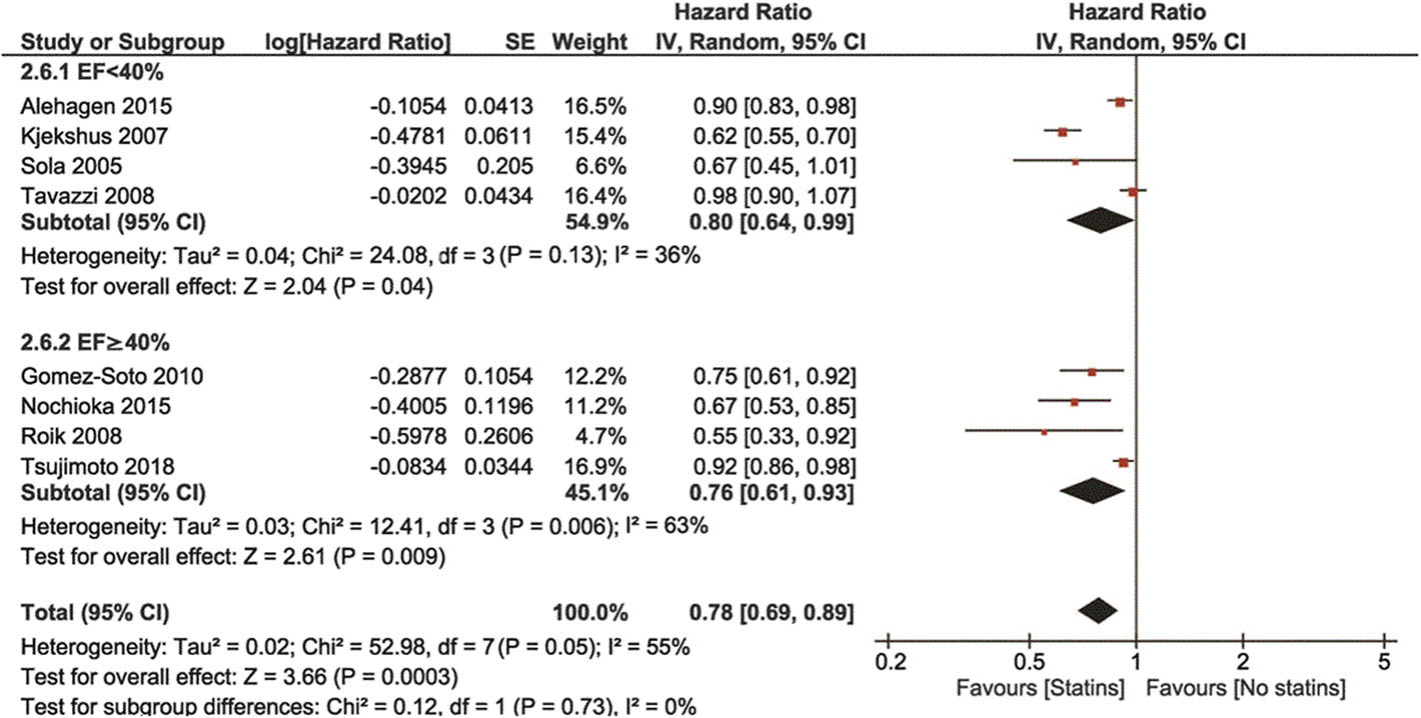
Association of statin versus non-statin use in HF patients with CV hospitalizations by LVEF value

In comparison to non-statin users, all-cause mortality was reduced in statin users in both EF < 40%and EF ≥40% groups (HR 0.77, 95% Cl: 0.68–0.86, *p* < 0.00001, and HR0.75, 95% Cl: 0.69–0.82, p < 0.00001, respectively, Fig. 4). CV mortality was also reduced in both EF groups using statins (HR 0.86, 95% Cl: 0.79–0.93, *p* = 0.0003, and HR 0.83, 95% Cl: 0.77–0.90, respectively, Fig. 5) with no differences between EF subgroups. Similar reduced were observed for CV hospitalizations – they were reduced in statin users in both EF groups (HR 0.80 95CI: 0.64–0.99, p = 0.04, and HR 0.76 95% CI: 0.61–0.93, *p* = 0.009, respectively, Fig. 6). Statin effects on all primary outcomes were confirmed when only perspective cohort studies were analyzed (after withdrawal of 2 RCTs) (Additional file 1: Figures. S1 to S6).

**Fig. 7:**
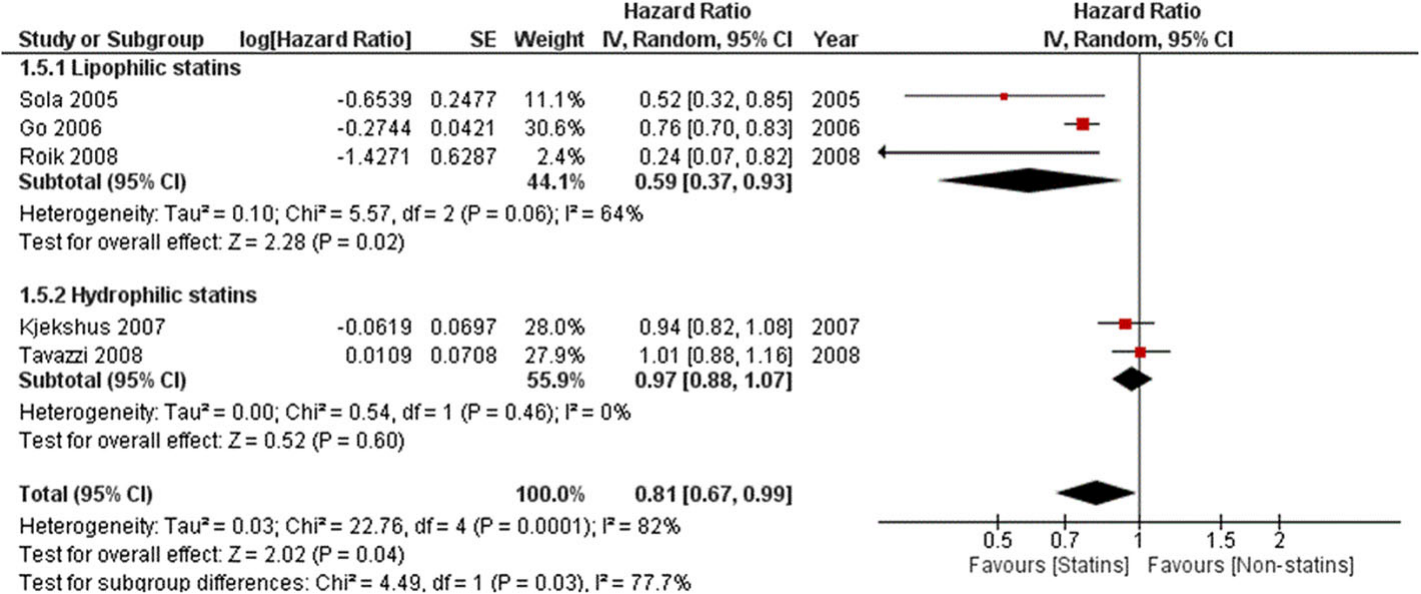
Association of statin versus non-statin use with all-cause mortality by type of liposolubility

**Fig. 8:**
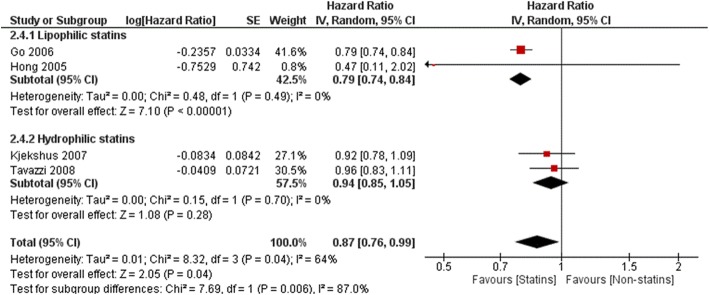
Association of statin versus non-statin use with CV mortality by type of liposolubility

**Fig. 9:**
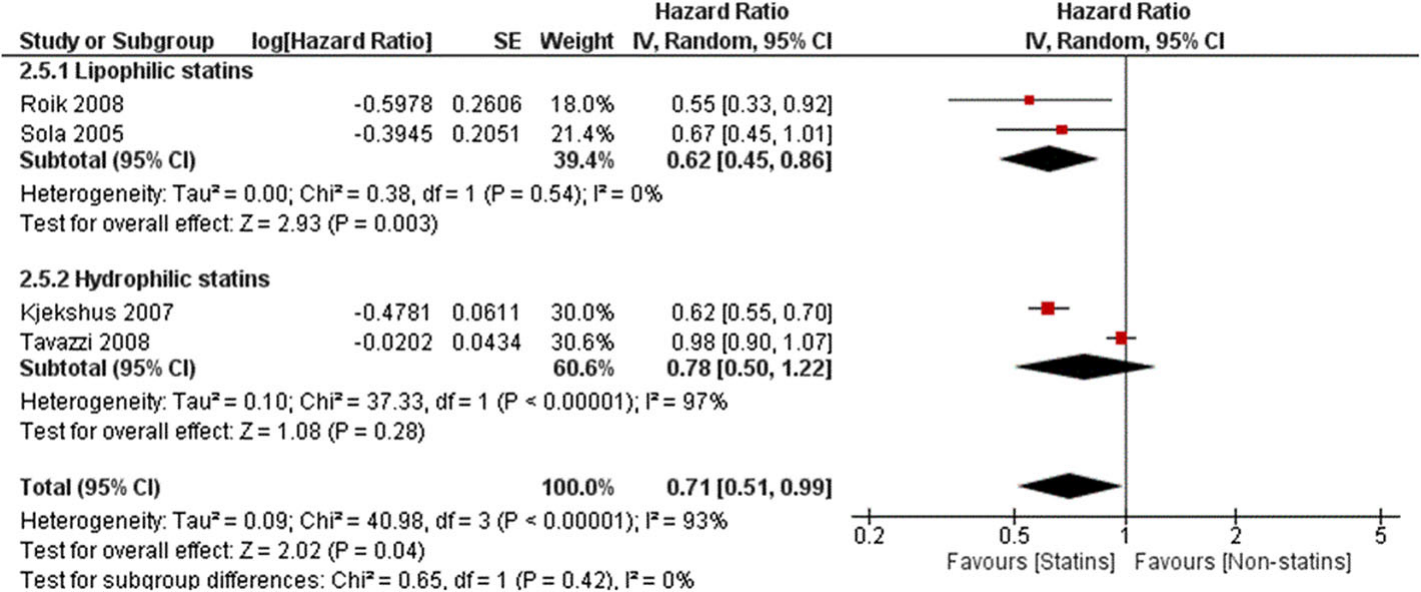
Association of statin versus non-statin use with CV hospitalization by type of liposolubility

Effect on all-cause mortality was higher for lipophilic compared to hydrophilic statins (HR 0.59, 95%Cl: 0.37–0.93, *p* = 0.02 and HR 0.97, 95%Cl: 0.88–1.07, p = 0.60, respectively, Fig. 7). Significant decreases of cardiovascular outcomes were also observed only with lipophilic statins- CV mortality (HR 0.79, 95%Cl: 0.74–0.88, *P* ≤ 0.00001 vs HR 0.94, 95% Cl: 0.85–1.05,*P* = 0.28, Fig. 8) and CV hospitalizations (HR 0.60, 95%Cl: 0.45–0.86, P = 0.003 vs HR 0.78, 95% Cl: 0.50–1.22, *P* = 0.28, Fig. 9).

### Risk of bias assessment.

The two included RCTs had low risk of bias (Additional file 1: Table S1). Many of the cohorts have good quality, about 20% of them that have fair quality (Additional file 1: Table S2).

## Discussion

This systematic review evaluated large cohort of HF patients from studies comparing the effect of statin therapy with non-statin therapy on clinical outcomes. Statin treatment decreased all-cause mortality, CV mortality and CV hospitalization in HF with either LVEF ≥40% or LVEF < 40%. Effects of statin use were similar in both EF groups, and also after excluding trials with randomization. Finally, lipophilic (e.g. atorvastatin) and no hydrophilic statins (e.g. rosuvastatin or pravastatin) showed significant reductions in clinical outcomes.

Statins are able to decrease vascular and myocardial oxidative stress [30, 31] and possess anti-inflammatory properties [32, 33]. A lot of available studies have shown that they limit signal transmission from membrane receptors and slow down pathologic heart and vessels remodeling, inhibit the action of angiotensin II, and process of apoptosis [31]. Statins might also change myocardial action potential plateau by modulation of Kv1.5 and Kv4.3 channels activity and inhibition of sympathetic nerve activity and in the consequence suppress arrhythmogenesis [34]. Those beneficial effects of statin therapy might be negated by increases in collagen turnover markers as well as a reduction in plasma coenzyme Q10 (CoQ10) levels in chronic heart failure (CHF) patients [35–37].

There has been a large discussion on the role of lipid-lowering therapy in HF patients. Available knowledge has indicated that statins might be potentially harmful in HF due to decreased endotoxin defense, diminishing thereby the potentially beneficial pleiotropic effects. There is some suggestive evidence that statins might reduce muscle strength and alter energy metabolism during aerobic exercise [38]. Based on our recent hypothesis this pro-sarcopenic effect of statins might be responsible of their limited efficacy in HF patients [38]. On the other hand, some available data indicate that statins may even have beneficial effects by preserving or even increasing lean mass and exercise performance [38]. Large trials with hydrophilic rosuvastatin did not indicate a significant role for statins in patients with chronic HF, although the drug did reduce the number of CV hospitalizations in the CORONA trial [39, 40]. Although the abovementioned RCTs using hydrophilic rosuvastatin showed no beneficial effect on all-cause mortality, other studies like Anker et al. [41] reported that patients with chronic HF in the Evaluation of Losastan In The Elderly-2 (ELITE 2) study who received statin therapy at baseline had lower mortality. The authors drew the conclusion that in chronic HF, treatment with statins was related to lower mortality, independent of cholesterol levels, disease etiology and clinical status [41, 42]. The results of our meta-analysis are in line with the above conclusions, as we also clearly showed significantly lower mortality in HF patients on statin therapy.

The significant decrease in CV hospitalization seen with rosuvastatin in the CORONA trial should not be overlooked [39, 40]. Based on the data of 5000 patients with ischemic HF, the authors concluded that the lack of statin benefits in the treatment of HF patients could have been associated with some specific patients’ characteristics [40]. Some criticism was associeted to the fact that the study participants were too old (mean age: 73 years); moreover, a large majority was in advanced HF stages [40]. In another important trial - the Effect of n-3 polyunsaturated fatty acids in patients with chronic heart failure (GISSI-HF), patients on statins were not included, which may have resulted in more patients with severe ischemia being excluded (individuals with ischemic HF represented only 40% of patients). Finally, patients receiving cardiac resynchronization therapy were either excluded or represented a small percentage of the studied population. It is important as there are some avaiable data, including a retrospective analysis of the Comparison of Medical Therapy, Pacing, and Defibrillation in Heart Failure (COMPANION) trial, suggesting that statin therapy might be associated with improved survival in HF patients receiving resynchronization therapy [43]. The GISSI-HF trial also had a relatively large number of patients who discontinued therapy for reasons other than adverse drug reactions (31%) compared with only 10% in the CORONA trial, raisingthe question of whether this might have impacted the final results of the study. The investigators of GISSI-HF also suggested that there were too few acute ischemic events in heart failure patients for a statin to show a benefit [39]. An alternative theory to explain the controversial results between real-life cohorts and the large RCTs was based on observation that in the CORONA trial the lowest N-terminal pro-B-type natriuretic peptide tertile did benefit from rosuvastatin therapy, with a significant reduction in the primary outcome. It has been suggested that in patients with less advanced HF, statin therapy might be benefitial in reduction of coronary events, whereas in severe HF, it is too late to for the potential benefits from statin therapy due to progressive loss of pump function [44].

The main finding from the meta-analysis of Preiss et al. [45] was a significant reduction in non-fatal MI and a modest (however still significant) reduction in first non-fatal HF hospitalizations [45]. The composite outcome of HF death and HF hospitalizations was also significantly reduced in the statin groups, but was driven exclusively by a reduction in HF hospitalizations. A noteworthy finding from the Preiss et al. study [45] concerns the mechanisms, by which statin therapy reduced the risk of HF hospitalizations. Interestingly, neither a reduced risk of non-fatal MI nor a decrease in LDL-C correlated with the risk of HF hospitalizations. These results raise the possibility that statins might have exerted beneficial effects on HF hospitalizations through their pleiotropic properties [45]. The results of our meta-analysis are consistent with the referred meta-analysis [45] and support a positive influence of the pleiotropic properties of statins on HF outcomes. What is worth emphasizing, recent evidence suggests that there is no class effect for statin use in the setting of HF, and we should expect different effects for hydrophilic and lipophylic statins [46].

It seems that one of the most important mechanisms of statins in this group of patients could be to rapidly affect signaling pathways in myocardial cell membranes and/or the autonomic nervous system, and in the consequence protecting them from life-threatening arrhythmias. The lipophilic statins (e.g. atorvastatin and simvastatin) become easily embedded in the cell membrane, having overlapping locations in the hydrocarbon core adjacent to the phospholipid head groups [47–49]. Evidence from a meta-analysis of RCTs by Lipinski et al. of statins in HF showed a significant benefit of hydrophilic atorvastatin on all-cause mortality, LVEF, and hospitalization due to HF, whereas similar effects were not observed in patients randomized to the hydrophilic rosuvastatin [50]. We have recently seen the same results for statin types as per our pro-sarcopenic hypothesis [38]. Our findings also support the findings by Liu et al. in patients with HF, which indicated a significant reduction in risk of all-cause mortality, CV mortality and hospitalization for worsening HF using lipophilic statins [50, 51]. Based on the available data it is known that lipophilic statins are to be much more susceptible to oxidative metabolism by the CYP450 system, and those metabolized by this system are more likely to produce muscle toxicity because of the risk of drug interactions with many drugs that inhibit CYP450 [38]. However based on the results of our study lipophilic statins revealed better outcomes in HF patients.

Recent ESC guidelines on HF have introduced a new phenotype based on LVEF, mid-range HF (HFmrEF) that falls between the classical HFrEF and HFpEF phenotypes [2, 7]. Therefore, statins might improve outcomes in these types of HF [53] through exerting beneficial effects on inflammation, LV hypertrophy, interstitial fibrosis, endothelial dysfunction and arterial stiffness, all of which contribute to the pathophysiology of HF with LVEF ≥40% [52, 54]. In the study of Alehagen et al. [55], 9140 patients in the prospective Swedish Heart Failure Registry with HF and EF ≥50% were divided into those treated with statins (*n* = 3427) and untreated with statins (n = 5713). Statins were associated with better one-year survival (85% vs 80%; *p* < 0.001), reduced CV death and composite all-cause mortality or CV hospitalization [55]. In a meta-analysis, Fukuta et al. [56] assessing the effect of statin therapy on mortality in patients with HF with LVEF≥45% with the use of propensity score analysis, showed that investigated therapy was associated with reduced mortality, which suggests the potential mortality benefit of statins in HFpEF [56]. Another meta-analysis included a total of 11 eligible studies with 17,985 patients with HF and EF > 45% [6]. Statin use was associated with a 40% lower risk of mortality (RR 0.60, 0.49–0.74, p < 0.001). Finally, cumulative meta-analysis by Liu et al. showed an obvious trend of reduction in mortality with statins [57, 58]. The results of our analysis in patients with HF and LVEF ≥40% are consistent with the results of Fukuta et al. and Liu et al. [56, 57].

There are some obvious limitations associated with this systematic review. First, there was limited information available on patient characteristics such as compliance with statin therapy or statin dosage. Included studies did not have enough data to check the correlations with cholesterol level and other variables like body mass index (BMI). The solubility of statins was a variable that was also not available in most of the trials, despite the fact the authors of this analysis asked all investigators of included studies about this; therefore, analyses by solubility was performed only based on limited number of studies with that information and hydrophilic statin arm included only two RCT studies while the other included only observational studies [59–61]. Most studies included in our meta-analysis were performed before 2016 when there was no fixed LVEF cut-off points for HFpEF and HFmrEF; that is why we combined HFpEF and HFmrEF patients in one group of patients with LVEF ≥40%. The HFmrEF patients, as a new and distinct group, had many intermediate characteristics compared with HFrEF and HFpEF subjects.

## Conclusions

Statins may have beneficial effect on main CV outcomes in HF patients irrespective of the different etiologies and EF levels. Lipophilic statins, and not hydrophilic statins might be favorable for patients with heart failure independently from their postulated prosarcopenic effects [38]. The present meta-analysis emphasizes the need for a new, well-design randomized study of the effect of statins, in particular lipophilic, in HF patients. There will also be a need for additional analyses assessing the impact of cholesterol levels, BMI, type and doses of statin, and body mass compartments on outcomes. This information could establish a target group of patients with HF who will benefit the most from statin therapy as well as the type and dose of statins that are optimal in these patients.

## Supplementary information

**Additional file 1: Appendix 1.** Assessment of risk of bias in the included studies using Cochrane criteria for RCTs. **Appendix 2.** Assessment of risk of bias in the included studies using Newcastle-Ottawa Quality Assessment Scale (NOS) for cohort studies. **Figure S1.** Association of statin versus non-statin use with all-cause mortality in heart failure only in cohort studies. **Figure. S2**. Association of statin versus non-statin use with CV mortality in heart failure only in cohort studies. **Figure S3.** Association of statin versus non-statin use hospitalization in heart failure only in cohort studies. **Figure S4.** Association of statin versus non-statin use with all-cause mortality by type of heart failure only in cohort studies. **Figure S5.** Association of statin versus non-statin use with CV mortality by type of heart failure only in cohort studies. **Figure S6.** Association of statin versus non-statin use with hospitalization by type of heart failure only in cohort studies. **Table S1.** Assessment of risk of bias in the included studies using Cochrane criteria for RCTs. **Table S2.** Assessment of risk of bias in the included studies using Newcastle-Ottawa Quality Assessment Scale (NOS) for cohort studies.

## Data Availability

All data generated or analysed during this study are included in this published article [and its supplementary information files.

## Abbreviations

ACC/AHA: American College of Cardiology/American Heart Association
BMI: body mass index
CAD: coronary artery disease
CHF: chronic heart failure
CI: confidence interval
CoQ10: coenzyme Q10
CV: cardiovascular
DM: diabetes mellitus
EAS: European Society of Atherosclerosis
HF: heart failure
HFmrEF: heart failure with mid-range left ventricular ejection fraction
HFpEF: heart failure with preserved ejection fraction
HFrEF: heart failure with reduced left ventricular ejection fraction
HR: hazard ratio
HTN: arterial hypertension
IRB: Institutional Review Board
LDL: low density cholesterol
LVEF: left ventricular ejection fraction
MI: myocardial infarction,
NLA: National Lipid Association
RCTs: randomized controlled trials
SD: standard deviation
SElogHR: standard errors of the log HR
TC: total cholesterol
TGs: triglycerides

## References

[1] Stone NJ, Robinson JG, Lichtenstein AH (2014). 2013 ACC/AHA guideline on the treatment of blood cholesterol to reduce atherosclerotic cardiovascular risk in adults: a report of the American College of Cardiology/American Heart Association task force on practice guidelines. Circulation.

[2] Ponikowski P, Voors AA, Anker SD, et al. ESC guidelines for the diagnosis and treatment of acute and chronic heart failure. Eur Heart J. 2016;37(27):2129–200. 10.1093/eurheartj/ehw128 27206819

[3] Owan TE, Hodge DO, Herges RM, Jacobsen SJ, Roger VL, Redfield MM (2006). Trends in prevalence and outcome of heart failure with preserved ejection fraction. N Engl J Med.

[4] Steinberg BA, Zhao X, Heidenreich PA (2012). Trends in patients hospitalized with heart failure and preserved left ventricular ejection fraction: prevalence, therapies, and outcomes. Circulation..

[5] Liao JK, Oesterle A. The Pleiotropic Effects of Statins - From Coronary Artery Disease and Stroke to Atrial Fibrillation and Ventricular Tachyarrhythmia. Curr Vasc Pharmacol. 2019;17(3):222–32. 10.2174/1570161116666180817155058 PMC6378117 30124154

[6] Moher D, Liberati A, Tetzlaff J, Altman DG, Group P (2009). Preferred reporting items for systematic reviews and meta-analyses: the PRISMA statement. BMJ..

[7] Ponikowski, P., Voors, A.A., Anker, S.D. et al. 2016 ESC guidelines for the diagnosis and treatment of acute and chronic heart failure. Rev Esp Cardiol (Engl Ed). 2016;69(12):1167. 10.1016/j.rec.2016.11.005 27894487

[8] Higgins JPT, Green S (editors). Cochrane Handbook for Systematic Reviews of Interventions Version 5.1.0 [updated March 2011]. The Cochrane Collaboration, 2011. Available from www.handbook.cochrane.org.

[9] Zeng X, Zhang Y, Kwong JS (2015). The methodological quality assessment tools for preclinical and clinical studies, systematic review and meta-analysis, and clinical practice guideline: a systematic review. J Evid Based Med.

[10] Cooper HM, Hedges LV (1994). The handbook of research synthesis.

[11] Hozo SP, Djulbegovic B, Hozo I (2005). Estimating the mean and variance from the median, range, and the size of a sample. BMC Med Res Methodol.

[12] Higgins JP, Thompson SG, Deeks JJ, Altman DG (2003). Measuring inconsistency in meta analyses. BMJ.

[13] Horwich TB, MacLellan WR, Fonarow GC (2004). Statin therapy is associated with improved survival in ischemic and non-ischemic heart failure. J Am Coll Cardiol.

[14] Sola S, Mir MQ, Rajagopalan S, Helmy T, Tandon N, Khan BV (2005). Statin therapy is associated with improved cardiovascular outcomes and levels of inflammatory markers in patients with heart failure. J Card Fail.

[15] Fukuta H, Sane DC, Brucks S, Little WC (2005). Statin therapy may be associated with lower mortality in patients with diastolic heart failure: a preliminary report. Circulation..

[16] Hong YJ, Jeong MH, Hyun DW (2005). Prognostic significance of simvastatin therapy in patients with ischemic heart failure who underwent percutaneous coronary intervention for acute myocardial infarction. Am J Cardiol.

[17] Go AS, Lee WY, Yangs J, Lo JC, Gurwitz JH (2006). Statin therapy and risks for death and hospitalization in chronic heart failure. JAMA..

[18] Kjekshus J, Apetrei E, Barrios V (2007). Rosuvastatin in older patients with systolic heart failure. N Engl J Med.

[19] HuanLoh P, Windram JD, Tin L (2007). The effects of initiation or continuation of statin therapy on cholesterol level and all-cause mortality after the diagnosis of left ventricular systolic dysfunction. Am Heart J.

[20] Coleman CI, Kluger J, Bhavnani S (2008). Association between statin use and mortality in patients with implantable cardioverter-defibrillators and left ventricular systolic dysfunction. Heart Rhythm.

[21] Roik M, Starczewska MH, Huczek Z, Kochanowski J, Opolski G (2008). Statin therapy and mortality among patients hospitalized with heart failure and preserved left ventricular function--a preliminary report. Acta Cardiol.

[22] Tavazzi L, Maggioni AP, Marchioli R (2008). Effect of rosuvastatin in patients with chronic heart failure (the GISSI-HF trial): a randomised, double-blind, placebo-controlled trial. Lancet..

[23] Gomez-Soto FM, Romero SP, Bernal JA (2010). Mortality and morbidity of newly diagnosed heart failure treated with statins: a propensity-adjusted cohort study. Int J Cardiol.

[24] Kaneko H, Suzuki S, Yajima J (2013). Clinical characteristics and long-term clinical outcomes of Japanese heart failure patients with preserved versus reduced left ventricular ejection fraction: a prospective cohort of Shinken database 2004-2011. J Cardiol.

[25] Yap J, Sim D, Lim CP (2015). Predictors of two-year mortality in Asian patients with heart failure and preserved ejection fraction. Int J Cardiol.

[26] Nochioka K, Sakata Y, Miyata S (2015). Prognostic impact of statin use in patients with heart failure and preserved ejection fraction. Circ J.

[27] Alehagen U, Benson L, Edner M (2015). Association between use of statins and mortality in patients with heart failure and ejection fraction of ≥50. Circ Heart Fail..

[28] Alehagen U, Benson L, Edner M (2015). Association between use of statins and outcomes in heart failure with reduced ejection fraction: prospective propensity score matched cohort study of 21 864 patients in the Swedish heart failure registry. Circ Heart Fail..

[29] Tsujimoto T, Kajio H (2018). Favorable effects of statins in the treatment of heart failure with preserved ejection fraction in patients without ischemic heart disease. Int J Cardiol.

[30] Brown JH, Del Re DP, Sussman MA (2006). The Rac and rho hall of fame: a decade of hypertrophic signaling hits. Circ Res.

[31] Wang CY, Liao JK (2007). Current advances in statin treatment: from molecular mechanisms to clinical practice. Arch Med Sci.

[32] Albert MA, Danielson E, Rifai N, Ridker PM; PRINCE Investigators. Effect of statin therapy on C-reactive protein levels: the pravastatin inflammation/CRP evaluation (PRINCE): a randomized trial and cohort study. JAMA. 2001;4;286(1): 64–70. 10.1001/jama.286.1.64 11434828

[33] Ray KK, Cannon CP, Cairns R (2005). PROVE IT-TIMI 22 investigators. Relationship between uncontrolled risk factors and C-reactive protein levels in patients receiving standard or intensive statin therapy for acute coronary syndromes in the PROVE IT-TIMI 22 trial. J Am Coll Cardiol.

[34] Tousoulis D, Oikonomou E, Siasos G, Stefanadis C (2014). Statins in heart failure--with preserved and reduced ejection fraction. An update. Pharmacol Ther..

[35] Ashton E, Windebank E, Skiba M (2011). Why did high-dose rosuvastatin not improve cardiac remodeling in chronic heart failure? Mechanistic insights from the UNIVERSE study. Int J Cardiol.

[36] Mazidi M, Kengne AP, Banach M (2018). Lipid and Blood Pressure Meta-analysis Collaboration Group. Effects of coenzyme Q10 supplementation on plasma C-reactive protein concentrations: A systematic review and meta-analysis of randomized controlled trials. Pharmacol Res.

[37] Banach M, Serban C, Ursoniu S (2015). Lipid and blood pressure Meta-analysis collaboration (LBPMC) group. Statin therapy and plasma coenzyme Q10 concentrations--a systematic review and meta-analysis of placebo-controlled trials. Pharmacol Res.

[38] Bielecka-Dabrowa A, Fabis J, Mikhailidis DP (2018). Prosarcopenic effects of statins may limit their effectiveness in patients with heart failure. Trends Pharmacol Sci.

[39] Tavazzi L, Maggioni AP, Marchioli R (2008). GISSI-HF investigators. Effect of rosuvastatin in patients with chronic heart failure (the GISSI-HF trial): a randomised, double-blind, placebo-controlled trial. Lancet.

[40] Rogers JK, Jhund PS, Perez AC (2014). Effect of rosuvastatin on repeat heart failure hospitalizations: the CORONA trial (controlled Rosuvastatin multinational trial in heart failure). JACC Heart Fail.

[41] Anker SD, Clark AL, Winkler R (2006). Statin use and survival in patients with chronic heart failure—results from two observational studies with 5200 patients. Int J Cardiol.

[42] Peter C, Westman, Michael J (2015). Lipinski the use of statins in patients with heart failure: more questions than answers. J Thorac Dis.

[43] Sumner AD, Boehmer JP, Saxon LA (2009). Statin use is associated with improved survival in patients with advanced heart failure receiving resynchronization therapy. Congest Heart Fail.

[44] Cleland JG, McMurray JJ, Kjekshus J (2009). Plasma concentration of amino-terminal pro-brain natriuretic peptide in chronic heart failure: prediction of cardiovascular events and interaction with the effects of rosuvastatin: a report from CORONA (Controlled Rosuvastatin Multinational Trial in Heart Failure). J Am Coll Cardiol.

[45] Preiss D, Campbell RT, Murray HM (2015). The effect of statin therapy on heart failure events: a collaborative meta-analysis of unpublished data from major randomized trials. Eur Heart J.

[46] Bytyçi I, Bajraktari G, Bhatt DL (2017). Lipid and blood pressure Meta-analysis collaboration (LBPMC) group. Hydrophilic vs lipophilic statins in coronary artery disease: a meta-analysis of randomized controlled trials. J Clin Lipidol.

[47] Wang JQ, Wu GR, Wang Z (2014). Long-term clinical outcomes of statin use for chronic heart failure: a meta-analysis of 15 prospective studies. Heart Lung Circ.

[48] Bonsu KO, Kadirvelu A, Reidpath DD (2013). Statins in heart failure: do we need another trial?. Vasc Health Risk Manag.

[49] Xian-zhi H, Sheng-hua Z, Xin-hong W (2010). The effect of early and intensive statin therapy on ventricular premature beat or non-sustained ventricular tachycardia in patients with acute coronary syndrome. Cardiol J.

[50] Lipinski MJ, Cauthen CA, Biondi-Zoccai GG (2009). Meta-analysis of randomized controlled trials of statins versus placebo in patients with heart failure. Am J Cardiol.

[51] Liu G, Zheng X-X, Xu Y-L (2014). Effects of lipophilic statins for heart failure: a meta-analysis of 13 randomised controlled trials. Heart, Lung, Circulation.

[52] Rauchhaus M, Coats AJ, Anker SD (2000). The endotoxin-lipoprotein hypothesis. Lancet.

[53] Bielecka-Dabrowa Agata, Mikhailidis Dimitri P, Hannam Simon, Aronow Wilbert S, Rysz Jacek, Banach Maciej (2011). Statins and dilated cardiomyopathy: do we have enough data?. Expert Opinion on Investigational Drugs.

[54] Zou C, Qi H, Liu ZH, Han L, Zhao C, Yang X (2013). Simvastatin activates the PPARγ-dependent pathway to prevent left ventricular hypertrophy associated with inhibition of RhoA signaling. Tex Heart Inst J.

[55] Alehagen U, Benson L, Edner M, Dahlström U, Lund LH (2015). Association between use of statins and mortality in patients with heart failure and ejection fraction of ≥50. Inhibitors of 3-hydroxy-3-methylglutaryl-coenzyme a (HMG-CoA) reductase occasionally cause myopathy characterized by weakness, pain, and elevated serum creatine phosphokinase (CK). Circ Heart Fail.

[56] Fukuta H, Goto T, Wakami K, Ohte N (2016). The effect of statins on mortality in heart failure with preserved ejection fraction: a meta-analysis of propensity score analyses. Int J Cardiol.

[57] Liu G, Zheng XX, Xu YL, Ru J, Hui RT, Huang XH (2014). Meta-analysis of the effect of statins on mortality in patients with preserved ejection fraction. Am J Cardiol.

[58] Dyrbuś K, Osadnik T, Desperak P, Desperak A, Gąsior M, Banach M (2018). Evaluation of dyslipidaemia and the impact of hypolipidemic therapy on prognosis in high and very high risk patients through the Hyperlipidaemia therapy in tERtiary Cardiological cEnTer (TERCET) registry. Pharmacol Res.

[59] Ursoniu S, Mikhailidis DP, Serban MC, Penson P, Toth PP, Ridker PM, Ray KK, Kees Hovingh G, Kastelein JJ, Hernandez AV, Manson JE, Rysz J, Banach M (2017). lipid and blood pressure Meta-analysis collaboration (LBPMC) group. The effect of statins on cardiovascular outcomes by smoking status: A systematic review and meta-analysis of randomized controlled trials. Pharmacol Res.

[60] Schachter M (2005). Chemical, pharmacokinetic and pharmacodynamic properties of statins: an update. Fundam Clin Pharmacol.

[61] Henninger C, Huelsenbeck S, Wenzel P, Brand M, Huelsenbeck J, Schad A, Fritz G (2015). Chronic heart damage following doxorubicin treatment is alleviated by lovastatin. Pharmacol Res.

