# Correction to: Association of statin use and clinical outcomes in heart failure patients: a systematic review and meta-analysis

**DOI:** 10.1186/s12944-020-01380-x

**Published:** 2020-09-20

**Authors:** Agata Bielecka-Dabrowa, Ibadete Bytyçi, Stephan Von Haehling, Stefan Anker, Jacek Jozwiak, Jacek Rysz, Adrian V. Hernandez, Gani Bajraktari, Dimitri P. Mikhailidis, Maciej Banach

**Affiliations:** 1 Department of Hypertension, Medical University of Lodz, Rzgowska, 281/289, 93-338 Łódź, Poland; 2 Department of Cardiology and Congenital Diseases of Adults, Polish Mother’s Memorial Hospital Research Institute (PMMHRI), Lodz, Poland; 3 Clinic of Cardiology, University Clinical Centre of Kosovo, Prishtina, Republic of Kosovo; 4 Department of Public Health and Clinical Medicine, Umeå University, Umeå, Sweden; 5 Department of Cardiology and Pneumology, University Medical Center Gottingen (UMG), Gottingen, Germany; 6 Charité-Universitätsmedizin Berlin, Berlin, Germany; 7 Department of Family Medicine and Public Health, Institute of Medicine, University of Opole, Opole, Poland; 8 Department of Nephrology, Hypertension and Family Medicine, Medical University of Lodz, Lodz, Poland; 9 Health Outcomes, Policy, and Evidence Synthesis (HOPES) Group, University of Connecticut School of Pharmacy, Storrs, CT USA; 10 School of Medicine, Universidad Peruana de Ciencias Aplicadas (UPC), Lima, Peru; 11 Department of Clinical Biochemistry, Royal Free Campus, University College London Medical School, University College London (UCL), London, UK

**Correction to: Lipids Health Dis 18, 188 (2019)**

**https://doi.org/10.1186/s12944-019-1135-z**

Following publication of the original article [1], the authors noticed a mistake in the surname of Prof. Dimitri P. Mikhailidis. In the published version, it is Mikhalidis and should be corrected to Mikhailidis.
