# Supplementary material for: Association of statin use and clinical outcomes in heart failure patients: a systematic review and meta-analysis
Source: Lipids Health Dis. 2019 Oct 31;18:188. doi: 10.1186/s12944-019-1135-z (PMC6822388; doi:10.1186/s12944-019-1135-z)
Supplement: Supplementary file 1 — Additional file 1: Appendix 1. Assessment of risk of bias in the included studies using Cochrane criteria for RCTs. Appendix 2. Assessment of risk of bias in the included studies using Newcastle-Ottawa Quality Assessment Scale (NOS) for cohort studies. Figure S1. Association of statin versus non-statin use with all-cause mortality in heart failure only in cohort studies. Figure. S2. Association of statin versus non-statin use with CV mortality in heart failure only in cohort studies. Figure S3. Association of statin versus non-statin use hospitalization in heart failure only in cohort studies. Figure S4. Association of statin versus non-statin use with all-cause mortality by type of heart failure only in cohort studies. Figure S5. Association of statin versus non-statin use with CV mortality by type of heart failure only in cohort studies. Figure S6. Association of statin versus non-statin use with hospitalization by type of heart failure only in cohort studies. Table S1. Assessment of risk of bias in the included studies using Cochrane criteria for RCTs. Table S2. Assessment of risk of bias in the included studies using Newcastle-Ottawa Quality Assessment Scale (NOS) for cohort studies. [file 12944_2019_1135_MOESM1_ESM.docx]

**SUPPLEMENTARY DATA**

**Appendix 1.Assessment of risk of bias in the included studies using Cochrane criteria for RCTs**

| **Study (trial) year** | **Sequence** | **Allocation** | **Blinding of** | **Blinding of** | **Incomplete** | **Selective** | **Other** |
| --- | --- | --- | --- | --- | --- | --- | --- |
|  | **generation** | **concealment** | **participants** | **outcome** | **outcome** | **outcome** | **bias** |
|  |  |  | **and personnel** | **assessment** | **data** | **reporting** |  |
| Kjekshus (CORONA) 2007 | L | L | L | U | L | L | L |
| Tevazzi (the GISSI-HF trial) 2008 | L | L | L | L | L | L | L |

Legend: L: low risk of bias; H: high risk of bias; U: unclear risk of bias.

**Appendix 2. Assessment of risk of bias in the included studies using Newcastle-Ottawa Quality Assessment Scale (NOS) for cohort studies**

| **Study, year** | **Selection** | **Selection** | **Selection** | **Selection** | **Comparability** | **Exposure** | **Exposure** | **Exposure** | **Quality** |
| --- | --- | --- | --- | --- | --- | --- | --- | --- | --- |
|  | **1** | **2** | **3** | **4** | **1** | **1** | **2** | **3** |  |
| Horwich et al. 2004 | a) | a) | a) | a) | a) | b) | a) | b) | Good |
| Sola et al. 2005 | b) | a) | a) | a) | b) | a), b) | a) | a) | Good |
| Fukuta et al 2005 | a) | a) | b) | a) | b) | b) | a) | b) | Good |
| Hong et al. 2005 | b) | a) | b) | a) | b) | b) |  | a) | Good |
| Go et al. 2006 | b) | a) | a) | b) | a) | b) | a) | b) | Good |
| Huan et al. 2007 | c) | a) | b) | a) | b) | d) | a) | b) | Good |
| Coleman et al. 2008 | b) | a) | d) | b) | b) | d) | a) | b) | Fair |
| Roik et al. 2008 | a) | a) | b) | b) | b) | b) | a) | a) | Good |
| Gomez-Soto et al. 2010 | a) | a) | c) | b) | b) | b) | a) | a) | Good |
| Kaneko et al. 2013 | c) | b) | c) | a) | b) | a) | a) | b) | Fair |
| Yap et al. 2015 | c) | a) | b) | a) | a) | b | d) | b) | Fair |
| Nochioka et al. 2015 | a) | a) | b) | a) | a) | b) | a) | b) | Good |
| Alehagen U et al.2015 | a) | a) | b) | a) | b) | a) | a) | b) | Good |
| Alehagen et al. 2015 | b) | a) | b) | a) | b) | a) | a) | b) | Good |
| Tsujimoto et al. 2018 | a) | b) | a) | a) | a) | a) | a) | b) | Good |

Legend: NOS: Selection- 1: a),b) one star, c), d) no star; Selection- 2: a) one star, b, c) no star; Selection- 3: a), b) one star, c), d), e) no star, Selection- 4: a) one star, b) no star; Comparability: a), b) one star, c) no star; Exposure-1: a),b) one star, c), d), e) no star; Exposure-2: a) one star, b) no star; Exposure-2**:** a),b) one star, c), d), no star.

**Good quality**: 3 or 4 stars in selection domain AND 1 or 2 stars in comparability domain AND 2 or 3 stars in outcome/exposure domain

**Fair quality:** 2 stars in selection domain AND 1 or 2 stars in comparability domain AND 2 or 3 stars in outcome/exposure domain

**Poor quality**: 0 or 1 star in selection domain OR 0 stars in comparability domain OR 0 or 1 stars in outcome/exposure domain

**Figure S1.** Association of statin versus non-statin use with all-cause mortality in heart failure only in cohort studies.


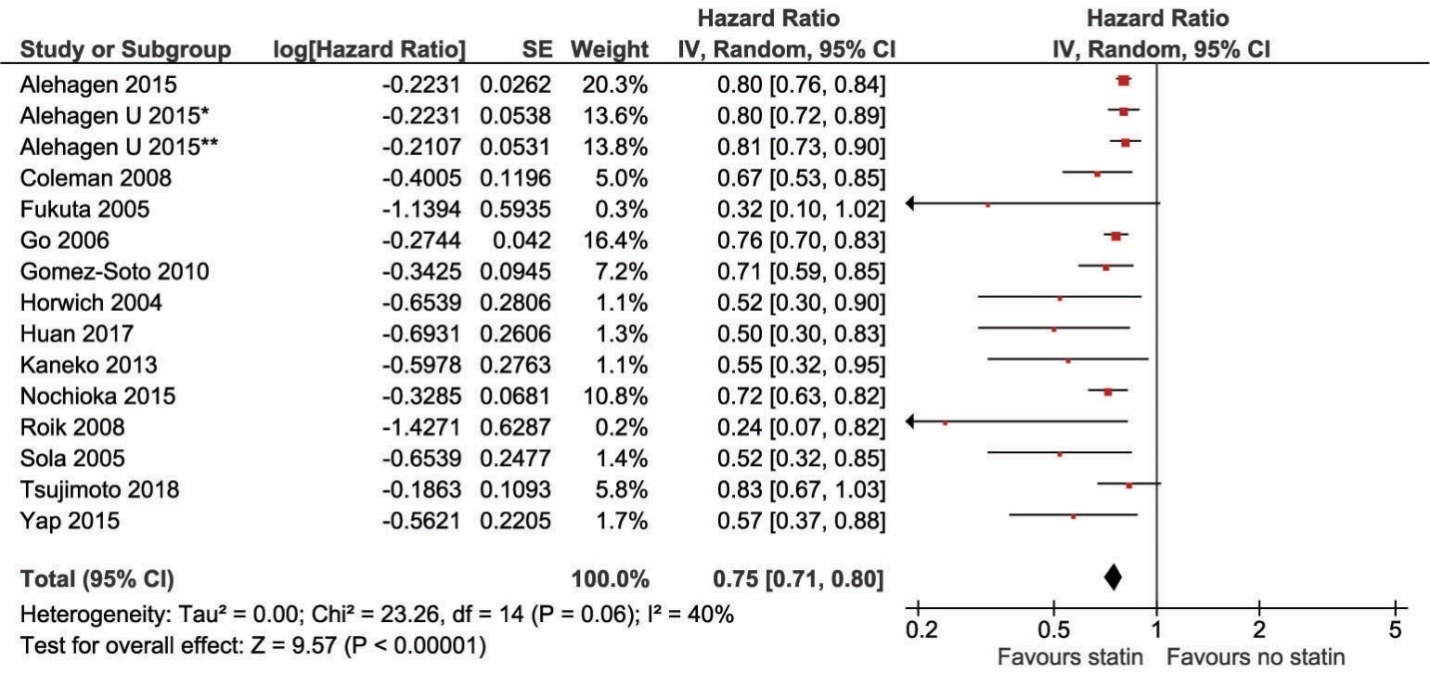


**Figure S2.** Association of statin versus non-statin use with CV mortality in heart failure only in cohort studies.


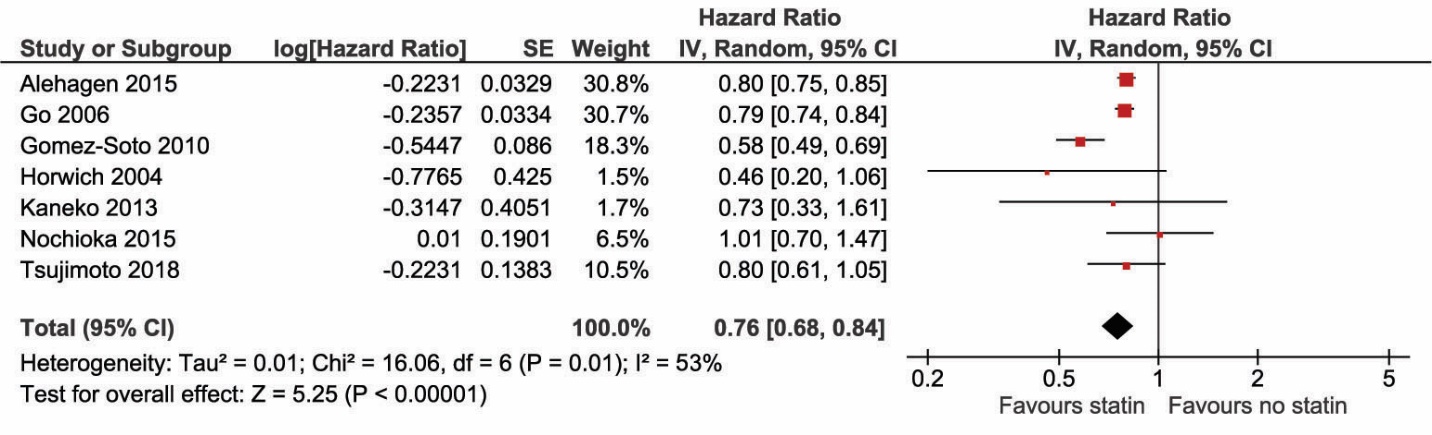


**Figure S3.** Association of statin versus non-statin use hospitalization in heart failure only in cohort studies.


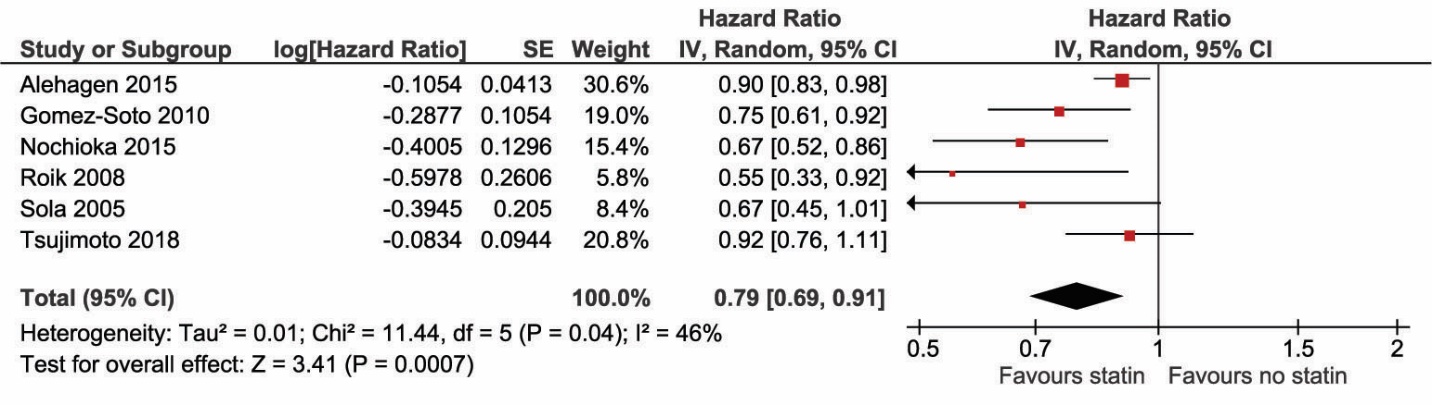


**Figure S4.** Association of statin versus non-statin use with all-cause mortality by type of heart failure only in cohort studies.

**
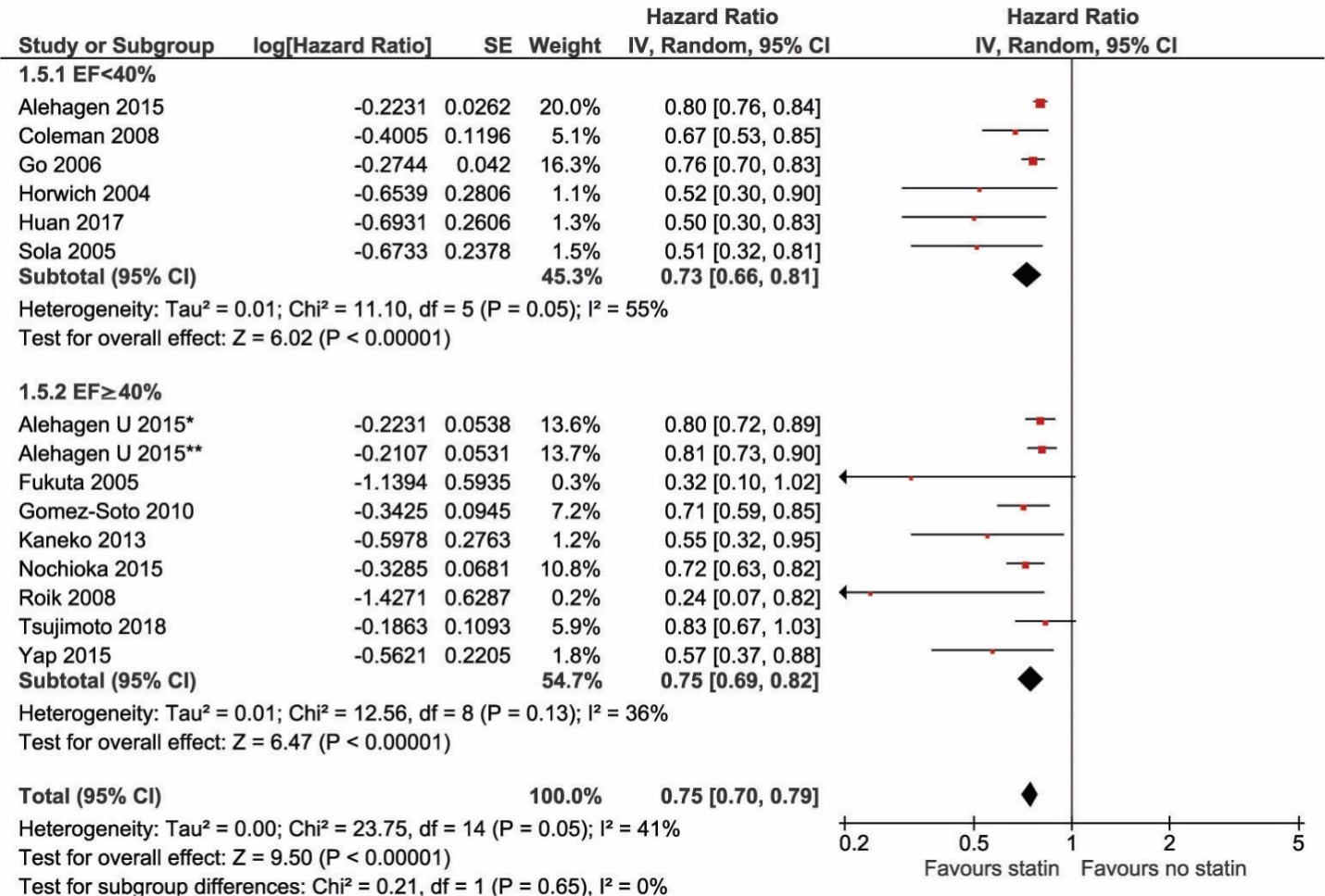
**

**Figure S5.** Association of statin versus non-statin use with CV mortality by type of heart failure only in cohort studies.


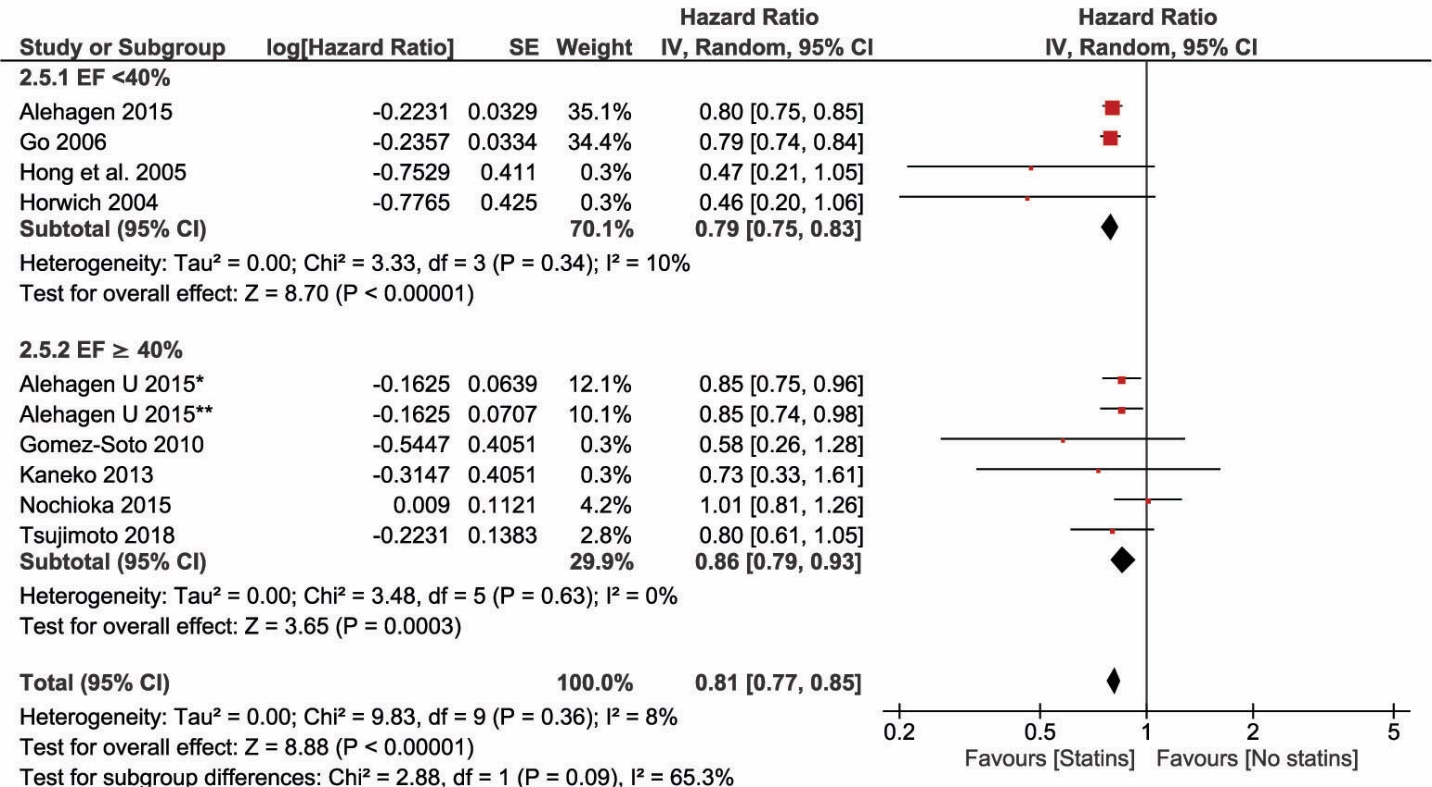


**Figure S6.** Association of statin versus non-statin use with hospitalization by type of heart failure only in cohort studies.


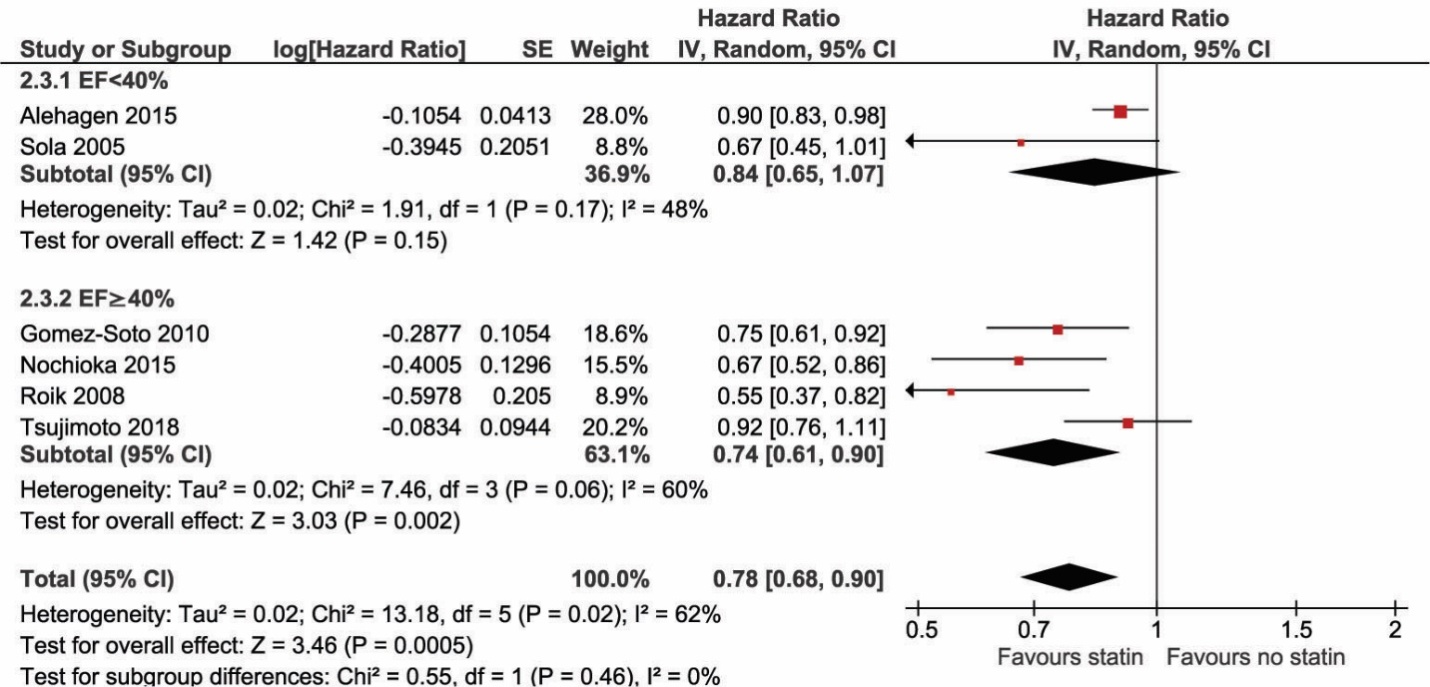


**Table S1.**Assessment of risk of bias in the included studies using Cochrane criteria for RCTs

| **Study (trial) year** | **Sequence** | **Allocation** | **Blinding of** | **Blinding of** | **Incomplete** | **Selective** | **Other** |
| --- | --- | --- | --- | --- | --- | --- | --- |
|  | **generation** | **concealment** | **participants** | **outcome** | **outcome** | **outcome** | **bias** |
|  |  |  | **and personnel** | **assessment** | **data** | **reporting** |  |
| Kjekshus (CORONA) 2007 | L | L | L | U | L | L | L |
| Tevazzi (the GISSI-HF trial) 2008 | L | L | L | L | L | L | L |

*Legend: L: low risk of bias; H: high risk of bias; U: unclear risk of bias.*

**Table S2.** Assessment of risk of bias in the included studies using Newcastle-Ottawa Quality Assessment Scale (NOS) for cohort studies.

| **Study, year** | **Selection** | **Selection** | **Selection** | **Selection** | **Comparability** | **Exposure** | **Exposure** | **Exposure** | **Quality** |
| --- | --- | --- | --- | --- | --- | --- | --- | --- | --- |
|  | **1** | **2** | **3** | **4** | **1** | **1** | **2** | **3** |  |
| Horwich et al. 2004 | a) | a) | a) | a) | a) | b) | a) | b) | Good |
| Sola et al. 2005 | b) | a) | a) | a) | b) | a), b) | a) | a) | Good |
| Fukuta et al 2005 | a) | a) | b) | a) | b) | b) | a) | b) | Good |
| Hong et al. 2005 | b) | a) | b) | a) | b) | b) |  | a) | Good |
| Go et al. 2006 | b) | a) | a) | b) | a) | b) | a) | b) | Good |
| Huan et al. 2007 | c) | a) | b) | a) | b) | d) | a) | b) | Good |
| Coleman et al. 2008 | b) | a) | d) | b) | b) | d) | a) | b) | Fair |
| Roik et al. 2008 | a) | a) | b) | b) | b) | b) | a) | a) | Good |
| Gomez-Soto et al. 2010 | a) | a) | c) | b) | b) | b) | a) | a) | Good |
| Kaneko et al. 2013 | c) | b) | c) | a) | b) | a) | a) | b) | Fair |
| Yap et al. 2015 | c) | a) | b) | a) | a) | b | d) | b) | Fair |
| Nochioka et al. 2015 | a) | a) | b) | a) | a) | b) | a) | b) | Good |
| Alehagen U et al.2015 | a) | a) | b) | a) | b) | a) | a) | b) | Good |
| Alehagen et al. 2015 | b) | a) | b) | a) | b) | a) | a) | b) | Good |
| Tsujimoto et al. 2018 | a) | b) | a) | a) | a) | a) | a) | b) | Good |

Legend: NOS: Selection- 1: a),b) one star, c), d) no star; Selection- 2: a) one star, b, c) no star; Selection- 3: a), b) one star, c), d), e) no star, Selection- 4: a) one star, b) no star; Comparability: a), b) one star, c) no star; Exposure-1: a),b) one star, c), d), e) no star; Exposure-2: a) one star, b) no star; Exposure-2**:** a),b) one star, c), d), no star.

**Good quality**: 3 or 4 stars in selection domain AND 1 or 2 stars in comparability domain AND 2 or 3 stars in outcome/exposure domain

**Fair quality:** 2 stars in selection domain AND 1 or 2 stars in comparability domain AND 2 or 3 stars in outcome/exposure domain

**Poor quality**: 0 or 1 star in selection domain OR 0 stars in comparability domain OR 0 or 1 stars in outcome/exposure domain
